# Association between dynamic estimated plasma volume trajectories and acute kidney injury after coronary artery bypass grafting

**DOI:** 10.3389/fmed.2026.1912347

**Published:** 2026-09-09

**Authors:** Beijun Gao, Hongmin Zhang, Ran An, Nan Cheng, Xin Wu, Xianqiang Wang, Juan Du

**Affiliations:** 1 Department of Surgical Intensive Care Unit, Fuwai Hospital, National Center for Cardiovascular Diseases, Chinese Academy of Medical Sciences and Peking Union Medical College, Beijing, China; 2 Department of Health Care, Peking Union Medical College Hospital, Chinese Academy of Medical Sciences and Peking Union Medical College, Beijing, China; 3 School of Biological Science and Medical Engineering, Beihang University, Beijing, China; 4 Department of Cardiovascular Surgery, State Key Laboratory of Cardiovascular Disease, Fuwai Hospital, National Center for Cardiovascular Diseases, Chinese Academy of Medical Sciences and Peking Union Medical College, Beijing, China

**Keywords:** acute kidney injury, central venous pressure, coronary artery bypass grafting, estimated plasma volume status, latent-class trajectory, venous congestion

## Abstract

**Background:**

Cardiac surgery-associated acute kidney injury (CSA-AKI) is driven partly by venous congestion, yet no single bedside measure fully captures volume status: central venous pressure (CVP) reflects venous pressure, not plasma-volume expansion. Whether dynamic estimated plasma volume status (ePVS), a non-invasive marker derived from routine hemoglobin and hematocrit, conveys AKI-relevant information independent of and complementary to CVP is unknown.

**Methods:**

In 4,818 adult patients with coronary artery bypass grafting (CABG) from Medical Information Mart for Intensive Care IV, version 3.0 (MIMIC-IV v3.0), serial ePVS over the first 48 h after admission to the intensive care unit (ICU) was modeled by latent-class linear mixed modeling to identify dynamic phenotypes. The primary outcome was Kidney Disease: Improving Global Outcomes (KDIGO) acute kidney injury (AKI) stage ≥ 2 within 72 h. Independence from CVP was tested by the ePVS-CVP correlation, progressive adjustment for admission CVP, CVP-stratified analysis with a trajectory by CVP interaction term, and parallel multiple-mediator analysis (24-h ΔHgb, peak CVP, net fluid balance). Forward-time landmark analyses and incremental-value metrics [Δ area under the curve (ΔAUC), net reclassification improvement (NRI), integrated discrimination improvement (IDI), and decision-curve analysis] were also performed.

**Results:**

Four phenotypes emerged: Low-stable (58.5%), Gradually-increasing (6.9%), Rapid-decline (8.7%), and High-stable (25.9%). Admission ePVS and CVP were essentially uncorrelated (Spearman *r* = 0.04), indicating non-redundant axes. Only the Gradually-increasing phenotype was independently associated with AKI (Model 3 OR 1.47, 95% CI 1.14 to 1.89), and this was barely changed by adjustment for admission CVP (from 1.58 in Model 1 to 1.49 in Model 2); peak CVP mediated only 9.9% of the total effect, the majority not being attributable to any single measured pathway. Forward-time landmarking amplified the association (OR 2.10 at 24 h; 2.26 at 48 h), arguing against reverse causation. The trajectory-by-CVP interaction was not significant (*p* = 0.20); the phenotype effect was numerically larger at low than high admission CVP (OR 1.76 versus 1.20), an exploratory pattern. Adding trajectory class to a static model produced modest gains (NRI 9.9%, IDI 0.4%, ΔAUC 0.003).

**Conclusion:**

Four distinct ePVS trajectories within 48 h after CABG carry differential AKI risk. A Gradually-increasing ePVS trajectory after CABG signifies AKI risk that is largely independent of and complementary to CVP, providing a non-invasive volume axis not captured by pressure-based monitoring.

## 1 Introduction

Cardiac surgery-associated acute kidney injury (CSA-AKI) occurs in 20 to 40% of patients after cardiac surgery (1, 2). In patients undergoing coronary artery bypass grafting (CABG), a meta-analysis of 33 randomized trials reported an incidence of 22.2% with cardiopulmonary bypass and 19.1% without (3). CSA-AKI independently predicts in-hospital mortality, prolonged mechanical ventilation, and chronic kidney disease (4). Its pathogenesis is multifactorial, integrating ischemia–reperfusion injury, inflammatory activation, neurohormonal stimulation, and the hemodilution and microcirculatory disturbance produced by cardiopulmonary bypass (2, 5). Renal venous congestion is increasingly recognized as a contributor, as elevated systemic venous pressure impairs renal perfusion independently of cardiac output (6). The role of postoperative fluid therapy, by contrast, remains difficult to quantify because the hemodynamic indices in routine use, including central venous pressure (CVP) and cumulative fluid balance, reflect the consequences of intravascular filling rather than plasma volume itself (7). A clinically interpretable marker of plasma volume status could complement these pressure-based metrics and help define hemodynamically distinct postoperative phenotypes with differential renal-injury risk.

Estimated plasma volume status (ePVS), calculated as (100 - Hct) / Hgb from routine hemoglobin and hematocrit using the Strauss formula (8), expresses plasma volume per gram of hemoglobin and is a non-invasive surrogate for intravascular hemodilution and plasma-volume expansion. In heart failure, ePVS independently predicts decompensation, rehospitalization, and all-cause mortality and is interpreted as a marker of subclinical congestion undetected by pressure-based monitoring (9). A similar role is reported in septic shock, where ePVS independently predicted 28-day mortality (10). In heart failure complicating myocardial infarction, plasma volume estimated by the Strauss formula predicts early cardiovascular events beyond routine clinical assessment, and an instantaneous estimate outperformed the change over time (11). However, a single admission value ignores the substantial within-patient change across the first 48 h after cardiopulmonary bypass, when hemodilution from priming fluid, transfusion, diuresis, and interstitial re-expansion act in opposing directions. Whether the ePVS trajectory, rather than a single snapshot, identifies clinically meaningful volume phenotypes with differential AKI risk is unknown.

Three issues remain unresolved. First, whether reproducible ePVS trajectory phenotypes exist within the first 48 h after CABG and confer differential AKI risk has not been formally examined. Second, and central to this study, it is unknown whether any trajectory-related risk is captured by CVP, the pressure-based index used at the bedside, or whether ePVS contributes information independent of and complementary to venous pressure. The candidate pathways linking a rising ePVS to renal injury have not been directly compared: venous congestion would act through venous pressure, hemodilution through falling hemoglobin, and cumulative volume overload through fluid balance. Third, the incremental value of dynamic over static ePVS measurement, in both statistical discrimination and clinical decision-making, has not been quantified. Accordingly, we identified dynamic ePVS trajectory phenotypes in a large cardiac surgical cohort, tested their association with subsequent AKI, and, centrally, determined whether any trajectory-related risk is independent of and complementary to CVP.

## 2 Methods

### 2.1 Data source and cohort

We used Medical Information Mart for Intensive Care IV, version 3.0 (MIMIC-IV v3.0), a publicly available critical-care database curated by the Massachusetts Institute of Technology and the Beth Israel Deaconess Medical Center (12). The author (B. G.) holds PhysioNet credentials (Record ID 12338471), and all identifiers were removed at source. Eligible patients underwent CABG and were admitted to the cardiac surgical intensive care unit (ICU) on the same calendar day; the first ICU admission per patient was retained. Exclusions were pre-existing chronic kidney disease [International Classification of Diseases (ICD)-9585.x or ICD-10 N18.x], renal replacement therapy before CABG, active pregnancy, and active malignancy. At least two paired Hgb/Hct measurements within 48 h were required for trajectory modeling. The final cohort comprised 4,818 patients (Supplementary Figure S1). Because MIMIC-IV is fully de-identified, ethical approval and informed consent were waived.

### 2.2 Estimated plasma volume status and central venous pressure

All Hgb and Hct measurements between 0 and 48 h after ICU admission were extracted, and pairs from the same blood draw were matched by chart time. ePVS was calculated by the Strauss formula:

ePVS(mL/g)=(100−Hct)/Hgb

With Hct as a percentage and Hgb in g/dL, the ratio gives milliliters of plasma per gram of hemoglobin. For window-level analyses, four target points were defined (0 to 6, 12, 24, and 48 h), and the ePVS measurement closest to each target was selected; the trajectory model used observed measurement times rather than these windowed values. CVP (chartevents itemids 220,074 and 223,774) was extracted over the same windows; the median 0 to 6 h value defined admission CVP and the maximum within 24 h defined peak CVP.

### 2.3 Outcome

The primary outcome was the first occurrence of Kidney Disease: Improving Global Outcomes (KDIGO) acute kidney injury stage ≥ 2 within 72 h of ICU admission, defined as a serum creatinine increase to ≥ 2.0 × baseline, urine output < 0.5 mL/kg/h for ≥ 12 h, or initiation of renal replacement therapy (13). Staging integrated creatinine and urine-output criteria; baseline creatinine was the latest preoperative value. The hour from ICU admission to the first qualifying threshold crossing served as the time-to-event variable for the landmark analyses.

### 2.4 Statistical analyses

Missing covariate values were imputed by predictive mean matching (R package mice), excluding the patient identifier from the predictor matrix; missingness was low, reaching at most 9.6% for the 48 h ePVS window (Supplementary Table S1). Covariates were imputed once rather than by multiple imputations, so the analysis does not propagate imputation uncertainty. A complete-case sensitivity analysis (*n* = 3,962) gave essentially the same result (Model 3 odds ratio 1.43 versus 1.47), which suggests the finding does not depend on how missing data were handled.

#### 2.4.1 Latent-class trajectory modeling

ePVS trajectories were identified by latent-class linear mixed modeling (14). Each class had its own quadratic fixed time effect with a within-class random intercept and no random slope. The median number of paired Hgb/Hct measurements per patient over 48 h was seven (interquartile range 6 to 8; range 2 to 27). Models with one to five classes were compared using a Bayesian information criterion, entropy, and minimum class proportion; the four-class solution was selected and refitted from 20 random-start grid search initializations. Each patient was assigned to the class with the highest posterior membership probability. The three- and five-class solutions, and the recurrence of a rising, AKI-enriched phenotype across them are reported in Supplementary Table S2.

#### 2.4.2 Primary regression and dose–response

The association between trajectory class (Low-stable as reference) and AKI ≥ stage 2 was modeled by logistic regression in three nested specifications. Model 1 included age, sex, body mass index, Sequential Organ Failure Assessment (SOFA) score, congestive heart failure, diabetes without complications, and chronic pulmonary disease. Model 2 added first-24 h mean arterial pressure, peak lactate, and admission CVP. Model 3 added 0-to 24-h red blood cell (RBC) transfusion volume and any noradrenaline use within 24 h. The models therefore adjust for early time-varying perioperative interventions (transfusion and vasopressor exposure) and hemodynamics, in addition to baseline demographic and comorbidity confounders, and not for baseline factors alone. Restricted cubic splines displayed non-linear dose–response relationships between continuous ePVS at each time point, admission CVP, and AKI risk. Covariates were selected *a priori* based on clinical relevance and previous evidence. Robustness to the depth of adjustment was examined in a sensitivity analysis (Supplementary Table S3). Given the multiple phenotype comparisons across the main and landmark analyses, *p*-values were additionally corrected by the Benjamini-Hochberg false-discovery-rate and Bonferroni methods (Supplementary Table S4).

#### 2.4.3 Independence from and complementarity with CVP

To test whether trajectory-related risk was independent of CVP, we (i) quantified the Spearman correlation between admission ePVS and admission CVP; (ii) compared phenotype effects before and after adding admission CVP (Model 1 vs. Model 2); (iii) repeated the Model 3 regression within strata of admission CVP (< 10 vs. ≥ 10 mmHg) and tested a trajectory by CVP interaction term; and (iv) decomposed the admission-ePVS effect through peak CVP in the mediation analysis (Section 2.4.4). The CVP-stratified analysis was pre-specified as exploratory.

#### 2.4.4 Mechanistic analysis: competing-pathway mediation

Three candidate mediators were tested in a parallel multiple-mediator counterfactual framework (15): the 24 h change in hemoglobin (ΔHgb), the peak CVP within 24 h, and the net intravenous fluid balance over 24 h. For each mediator M, we fitted a linear model of M on admission ePVS and the Model 3 covariates (the a-path) and a logistic model of AKI on M, admission ePVS, and the same covariates (the b-path). The natural indirect effect was estimated as a × b on the logit scale, with 95% non-parametric percentile-bootstrap confidence intervals from 1,500 resamples. This mediation analysis is exploratory and assumes no unmeasured mediator-outcome confounding; because ePVS is a deterministic function of hemoglobin, the change-in-hemoglobin pathway is not statistically identifiable, and proportions mediated are reported with bootstrap confidence intervals in Supplementary Table S5.

#### 2.4.5 Forward-time validation: landmark analyses

To address reverse causation, landmark analyses were performed at 24 h and 48 h (16). At each landmark T, patients with AKI before T were excluded and the event was redefined as AKI between T and 72 h. Trajectory-class effects were re-estimated with logistic regression and left-truncated Cox proportional-hazards models.

#### 2.4.6 Incremental value

A static-only model (admission ePVS plus Model 3 covariates) was compared with a dynamic-augmented model (the same plus trajectory class) by the change in C-statistic (ΔAUC), continuous net reclassification improvement (NRI), integrated discrimination improvement (IDI), and decision-curve analysis across threshold probabilities 0.05 to 0.65, with 95% confidence intervals (CIs) from 1,500 bootstrap replicates.

### 2.5 Software

Analyses used R 4.5.2 (packages lcmm, rms, mice, pROC; latent-class modeling, regression, and restricted cubic splines) and Python 3.12 (mediation, landmark, and NRI/IDI/DCA).

## 3 Results

### 3.1 Cohort and trajectory phenotypes

**Figure 1:**
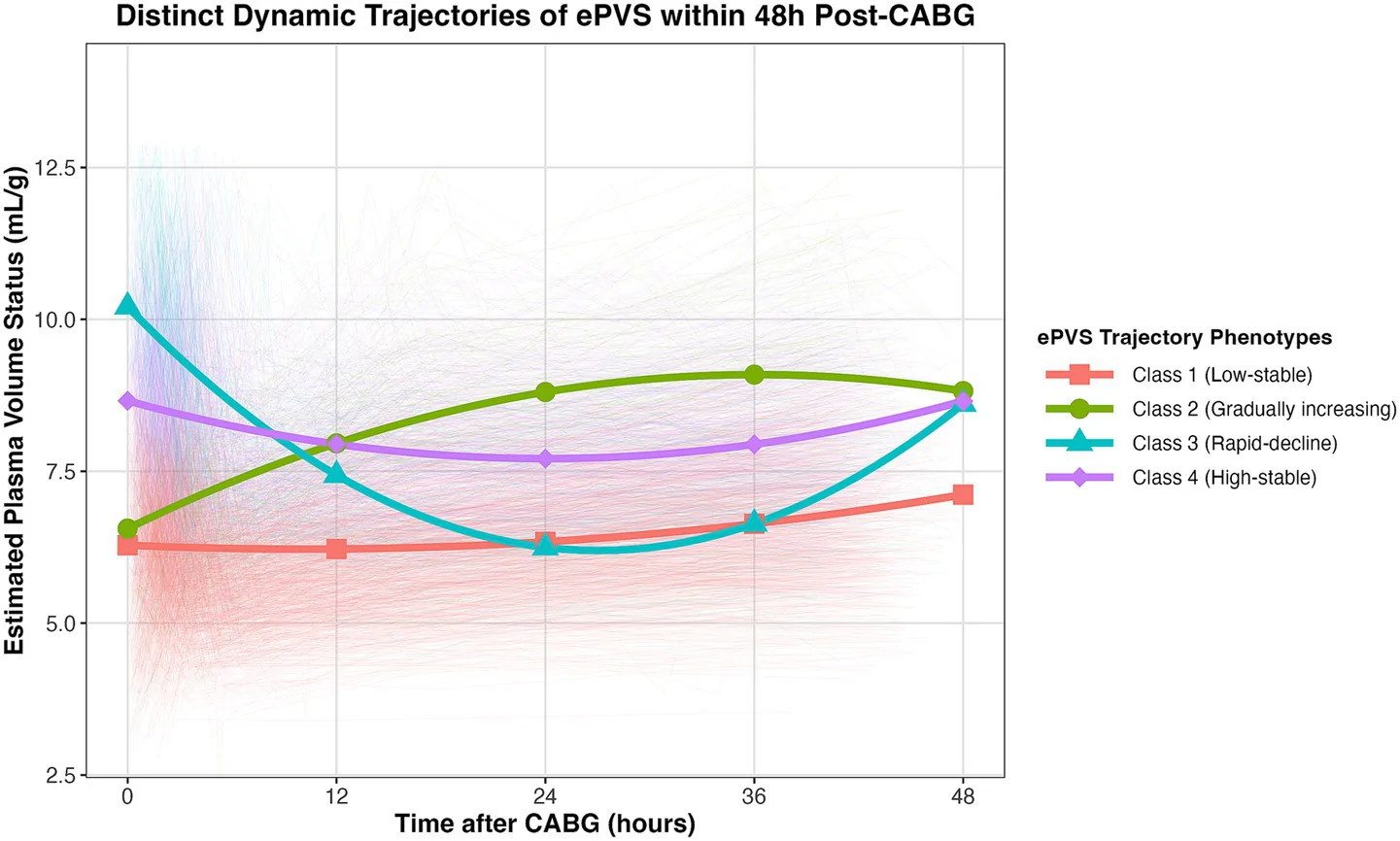
Four estimated plasma volume status (ePVS) trajectories within 48 h of intensive care unit (ICU) admission after coronary artery bypass grafting (CABG), identified by latent-class mixed modeling. Bold lines show class-mean predicted trajectories; thin background lines show individual observations.

A total of 4,818 patients were included (Supplementary Figure S1). The mean age was 67.8 ± 10.0 years, and 78.7% were male; 40.1% developed AKI stage ≥ 2 within 72 h. Four ePVS trajectory phenotypes were identified (Figure 1). The Low-stable phenotype was the largest (*n* = 2,819; 58.5%), with admission ePVS 6.28 mL/g remaining near 6.2 to 7.1 mL/g over 48 h (AKI 39.6%). The Gradually-increasing phenotype (*n* = 332; 6.9%) rose from 6.56 mL/g at admission to a peak of 9.09 mL/g at 36 h and had the highest AKI rate (47.0%). The Rapid-decline phenotype (*n* = 417; 8.7%) had the highest admission ePVS (10.21 mL/g), which fell to 6.25 mL/g at 24 h and then rebounded to 8.61 mL/g at 48 h (AKI 40.3%); this group received the largest median RBC transfusion in the first 24 h (700 mL). The High-stable phenotype (*n* = 1,250; 25.9%) maintained an admission ePVS of 8.66 mL/g, oscillating narrowly between 7.7 and 8.7 mL/g (AKI 39.4%). The phenotypes differed markedly in sex distribution, from 7.2% female in Low-stable to 61.6% in Rapid-decline, which follows from the presence of hemoglobin in the denominator of ePVS.

Baseline characteristics by phenotype are shown in Table 1. Rapid-decline patients had the highest prevalence of congestive heart failure (31.4%) and chronic pulmonary disease (26.1%) and the longest mechanical ventilation (median 11 h). Gradually-increasing patients had the highest first-24-h net intravenous fluid balance (median 9,050 mL) and the highest noradrenaline use (16.9%).

**Table 1:** Baseline characteristics by ePVS trajectory phenotype.

Variable	Low-stable (*n* = 2,819)	Gradually-increasing (*n* = 332)	Rapid-decline (*n* = 417)	High-stable (*n* = 1,250)	*p*-value
Demographics					
Age (years)	65.65 (9.62)	67.41 (9.86)	73.60 (9.40)	70.86 (9.61)	<0.001
Gender (Male, %)	2,615 (92.8)	275 (82.8)	160 (38.4)	742 (59.4)	< 0.001
Body Mass Index (kg/m2)	30.81 (5.53)	29.60 (5.75)	28.73 (5.94)	29.11 (5.62)	<0.001
Disease severity at admission					
SOFA score at admission	5.00 [3.00, 6.00]	5.00 [3.00, 7.00]	5.00 [4.00, 7.00]	5.00 [3.00, 7.00]	<0.001
Comorbidities					
Congestive heart failure (%)	434 (15.4)	65 (19.6)	131 (31.4)	271 (21.7)	<0.001
Diabetes (%)	851 (30.2)	104 (31.3)	162 (38.8)	454 (36.3)	<0.001
Myocardial infarction (%)	1,004 (35.6)	124 (37.3)	162 (38.8)	468 (37.4)	0.474
Chronic pulmonary disease (%)	408 (14.5)	63 (19.0)	109 (26.1)	241 (19.3)	<0.001
Vital signs (first 24 h)					
Heart rate (bpm)	82.71 (8.88)	84.04 (9.40)	82.54 (8.16)	82.65 (8.44)	0.053
Mean arterial pressure (mmHg)	75.06 (5.08)	72.68 (6.02)	73.20 (5.46)	73.39 (5.46)	<0.001
Body temperature (Celsius)	36.76 (0.36)	36.75 (0.35)	36.67 (0.41)	36.65 (0.36)	<0.001
Laboratory (first 24 h)					
Peak creatinine (mg/dL)	0.90 [0.80, 1.00]	0.90 [0.80, 1.10]	0.90 [0.70, 1.00]	0.90 [0.70, 1.00]	<0.001
Peak blood urea nitrogen (mg/dL)	16.00 [13.00, 19.00]	16.00 [13.00, 19.00]	16.00 [13.00, 21.00]	16.00 [12.00, 19.00]	0.003
Peak sodium (mmol/L)	139.00 [137.00, 141.00]	139.00 [138.00, 141.00]	140.00 [138.00, 142.00]	139.00 [137.00, 141.00]	<0.001
Peak lactate (mmol/L)	2.40 [1.90, 3.00]	2.70 [2.20, 3.80]	2.90 [2.40, 3.70]	2.60 [2.10, 3.40]	<0.001
Maximum anion gap (mmol/L)	12.00 [10.00, 14.00]	12.00 [10.00, 14.00]	12.00 [10.00, 14.00]	12.00 [10.00, 14.00]	0.022
Maximum INR	1.40 [1.30, 1.50]	1.50 [1.30, 1.60]	1.50 [1.40, 1.70]	1.50 [1.30, 1.60]	<0.001
Minimum fibrinogen (mg/dL)	212.00 [178.00, 256.00]	195.00 [166.75, 239.00]	181.00 [149.00, 226.00]	195.00 [164.00, 238.00]	<0.001
ICU course (first 24 h)					
Ventilation duration (hours)	5.28 [2.00, 9.92]	8.00 [3.98, 14.58]	11.00 [6.00, 19.00]	7.00 [3.67, 13.00]	<0.001
Norepinephrine use (%)	240 (8.5)	56 (16.9)	61 (14.6)	141 (11.3)	<0.001
Net IV fluid balance 0-24 h (mL)	7479.92 [5087.97, 9481.62]	9050.26 [6359.65, 11584.97]	7904.79 [5836.82, 9953.74]	7836.53 [5440.71, 9789.79]	<0.001
Urine output 0-24 h (mL)	1920.00 [1465.50, 2470.00]	1677.50 [1222.00, 2193.75]	1770.00 [1405.00, 2428.00]	1790.00 [1365.50, 2435.00]	<0.001
RBC transfusion 0-24 h (mL)	0.00 [0.00, 0.00]	0.00 [0.00, 65.25]	700.00 [350.00, 1050.00]	0.00 [0.00, 350.00]	<0.001
Outcomes					
Severe AKI (Stage 2/3, %)	1,116 (39.6)	156 (47.0)	168 (40.3)	493 (39.4)	0.072
28-day Mortality (%)	14 (0.5)	4 (1.2)	8 (1.9)	11 (0.9)	0.011

Estimated plasma volume status (ePVS); Sequential Organ Failure Assessment (SOFA); international normalized ratio (INR); intensive care unit (ICU); red blood cell (RBC); acute kidney injury (AKI).

### 3.2 The gradually-increasing phenotype carries the highest adjusted AKI risk

**Figure 2:**
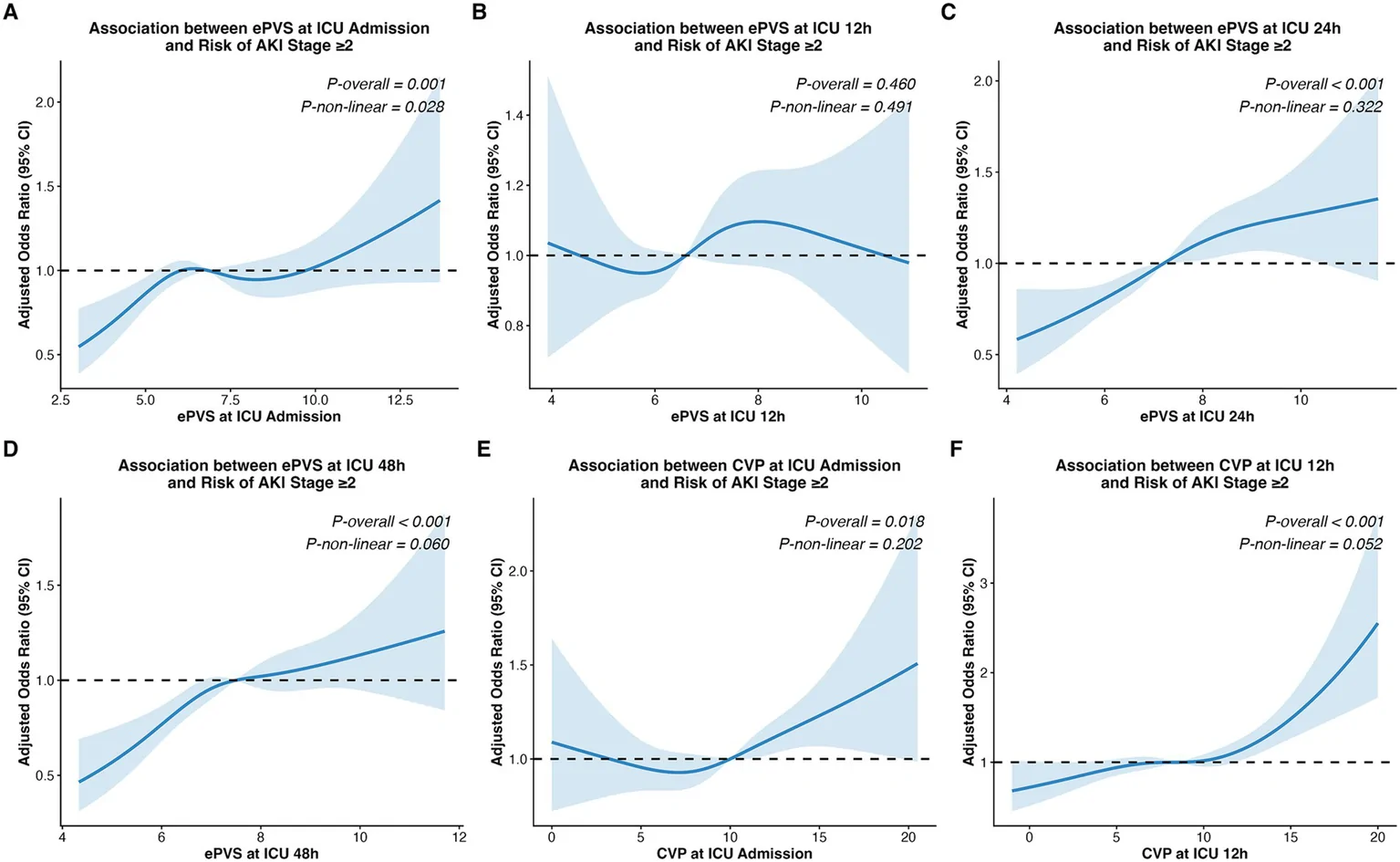
Restricted cubic spline curves of the adjusted odds ratios for acute kidney injury (AKI) stage ≥ 2 according to estimated plasma volume status (ePVS; mL/g) at intensive care unit (ICU) admission and at 12, 24, and 48 h **(A–D)**, and central venous pressure (CVP; mmHg) at ICU admission and 12 h **(E,F)**. Shaded bands show 95% confidence intervals; dashed lines indicate an odds ratio of 1.0. P-overall and P-nonlinear test the overall and nonlinear associations, respectively.

Across three nested logistic models, the Gradually-increasing phenotype was consistently associated with elevated AKI risk relative to Low-stable (Table 2). The adjusted OR was 1.58 (95% CI 1.23 to 2.02; *p* < 0.001) in Model 1, 1.49 (1.16 to 1.91; *p* = 0.002) in Model 2 after adjustment for early hemodynamics, including CVP, and 1.47 (1.14 to 1.89; *p* = 0.003) in Model 3. This association was already evident without adjustment (crude OR 1.35, 95% CI 1.08 to 1.70, *p* = 0.010), was stable across the depth of adjustment (Supplementary Table S3), and remained significant after correction for multiple comparisons (Benjamini-Hochberg *q* = 0.012, Bonferroni-corrected *p* = 0.024; Supplementary Table S4). Neither the Rapid-decline phenotype (Model 3: OR 0.85, 95% CI 0.64–1.11) nor the High-stable phenotype (OR 0.96, 95% CI 0.81–1.13) differed significantly from the Low-stable phenotype. Restricted cubic splines (Figures 2A–D) showed a monotonic dose–response between continuous ePVS and the adjusted log-odds of AKI at every time window, with an inflection near 7 mL/g and no plateau. This inflection describes the shape of the continuous dose–response relationship rather than a diagnostic threshold. Consistent with this, neither quintiles of admission ePVS, a fixed 7 mL/g cut-off, nor the 0 to 24 h or 0 to 48 h change stratified AKI risk (Supplementary Table S7). Admission and 12-h CVP showed a J-shaped relationship with AKI (nadir near 8 to 10 mmHg; Figures 2E,F), supporting the 10 mmHg cut-off used below.

**Table 2:** Adjusted odds ratios for AKI stage ≥ 2 by ePVS trajectory phenotype across three nested logistic regression models; Low-stable is the reference phenotype.

ePVS trajectory phenotype	Model 1 OR (95% CI), *p*	Model 2 OR (95% CI), *p*	Model 3 OR (95% CI), *p*
Low-stable	Reference	Reference	Reference
Gradually-increasing	1.58 (1.23 to 2.02), <0.001	1.49 (1.16 to 1.91), 0.002	1.47 (1.14 to 1.89), 0.003
Rapid-decline	1.00 (0.77 to 1.29), 0.992	0.93 (0.72 to 1.20), 0.572	0.85 (0.64 to 1.11), 0.228
High-stable	1.04 (0.88 to 1.22), 0.665	0.98 (0.83 to 1.16), 0.842	0.96 (0.81 to 1.13), 0.600
Model AUC	0.714 (95% CI 0.699 to 0.728)	0.720 (95% CI 0.705 to 0.734)	0.721 (95% CI 0.707 to 0.736)

Model 1: age, sex, BMI, SOFA, congestive heart failure, diabetes without complications, chronic pulmonary disease.

Model 2: plus first-24-h mean arterial pressure, peak lactate, admission CVP.

Model 3: plus 0 to 24 h RBC transfusion volume and any noradrenaline use within 24 h. AUC, area under the receiver-operating-characteristic curve.

Acute kidney injury (AKI); estimated plasma volume status (ePVS); area under the receiver-operating-characteristic curve (AUC); odds ratio (OR); confidence interval (CI); body mass index (BMI); Sequential Organ Failure Assessment (SOFA); central venous pressure (CVP); red blood cell (RBC).

### 3.3 The ePVS-AKI association is independent of and complementary to CVP

**Figure 3:**
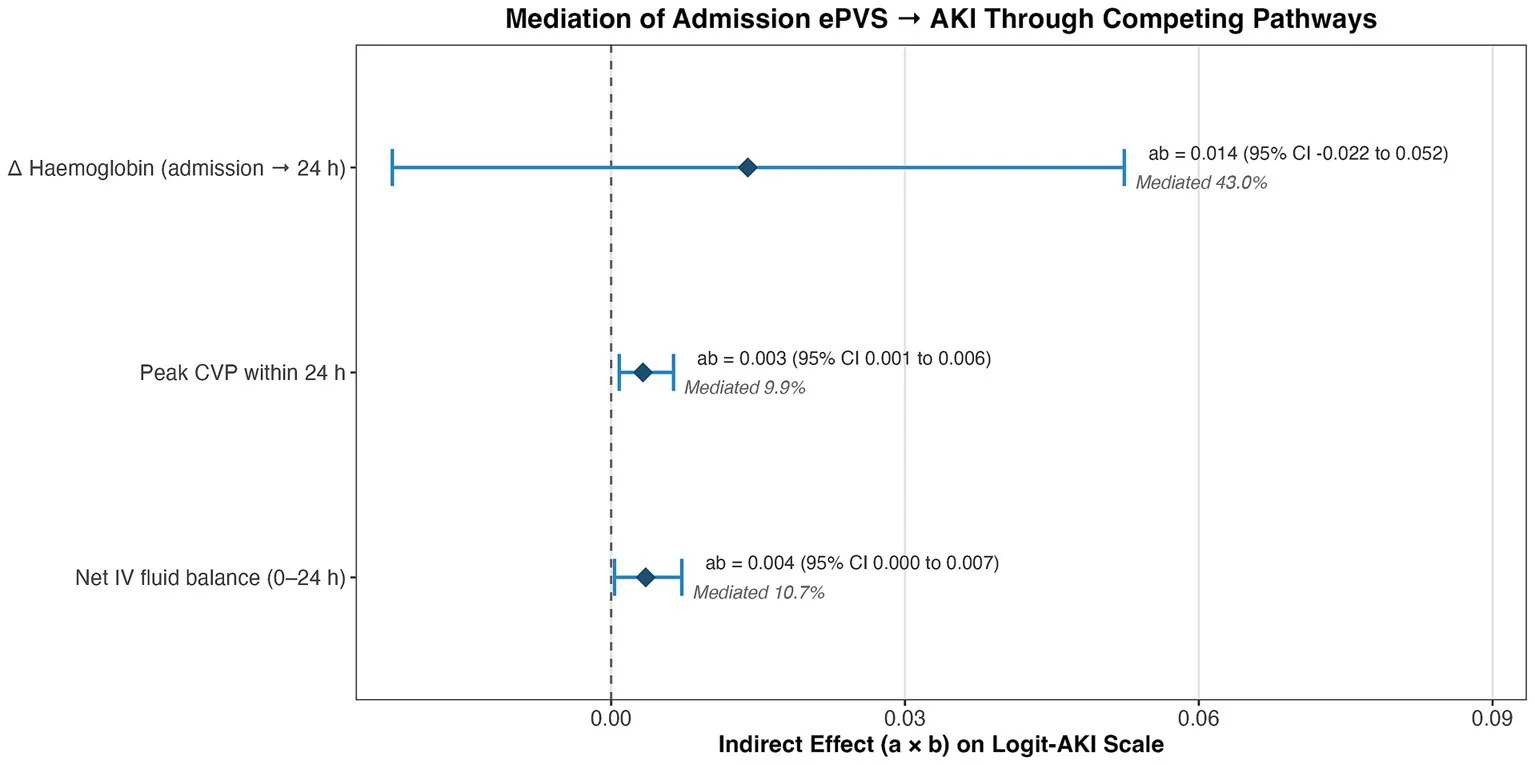
Competing-pathway mediation of the admission estimated plasma volume status (ePVS) effect on acute kidney injury (AKI) through three parallel pathways: hemodilution (ΔHgb 0 to 24 h), venous congestion (peak CVP within 24 h), and volume overload (net IV fluid balance 0 to 24 h). Points are bootstrap-mean indirect effects (a × b) on the logit-AKI scale, with 95% percentile confidence intervals (CIs).

Admission ePVS and admission CVP were essentially uncorrelated (Spearman *r* = 0.04; Supplementary Figure S2), indicating that the two indices capture distinct dimensions of volume status, namely plasma volume or hemodilution and venous pressure, rather than a single construct. Consistent with this, the Gradually-increasing phenotype association with AKI was barely attenuated by adjustment for admission CVP (from 1.58 in Model 1 to 1.49 in Model 2). In the parallel multiple-mediator analysis, peak CVP within 24 h accounted for only 9.9% of the total effect (Figure 3; Table 3); even after summing all three candidate pathways, together, the two statistically robust pathways, peak CVP and net fluid balance, accounted for about 21%, leaving the majority of the ePVS effect unexplained by venous pressure or fluid balance. The ΔHgb pathway had a larger point estimate (43.0%) but is not statistically identifiable because ePVS is a deterministic function of hemoglobin and is therefore not interpreted.

**Table 3:** Decomposition of the admission ePVS effect on AKI stage ≥ 2 through three parallel candidate mediators (counterfactual parallel multiple-mediator analysis; *n* = 4,758).

Mediator (M)	a-path (ePVS to M)	b-path (M to AKI, given ePVS)	Indirect a × b (95% CI)	Proportion mediated	Significance
ΔHgb (0 to 24 h)	*β* = 0.745, *p* < 0.001	*β* = 0.019, *p* = 0.43	0.014 (−0.022 to 0.052)	43.0%	Not significant (CI crosses zero)
Peak CVP (0 to 24 h)	*β* = 0.236, *p* < 0.001	*β* = 0.014, *p* = 0.005	0.003 (0.001 to 0.006)	9.9%	Significant
Net IV fluid balance (0 to 24 h)	*β* = 66.97, *p* = 0.041	*β* = 5.3 × 10^−5^, *p* < 0.001	0.004 (0.0003 to 0.0072)	10.7%	Significant

Total (unmediated) effect of admission ePVS on AKI on the logit scale = 0.033. The two statistically robust pathways (peak CVP and net fluid balance) together accounted for about 21% of this effect (indirect effects 0.003 + 0.004 = 0.007); the Δ Hgb pathway had a larger point estimate (about 43%) but a confidence interval crossing zero and is not statistically robust. The majority of the ePVS effect was therefore not attributable to any of these three measured pathways. a-paths from OLS; b-paths from logistic regression adjusting for the Model 3 covariates; 95% CIs from 1,500 percentile-bootstrap resamples.

Estimated plasma volume status (ePVS); acute kidney injury (AKI); confidence interval (CI); central venous pressure (CVP); ordinary least squares (OLS).

**Figure 4:**
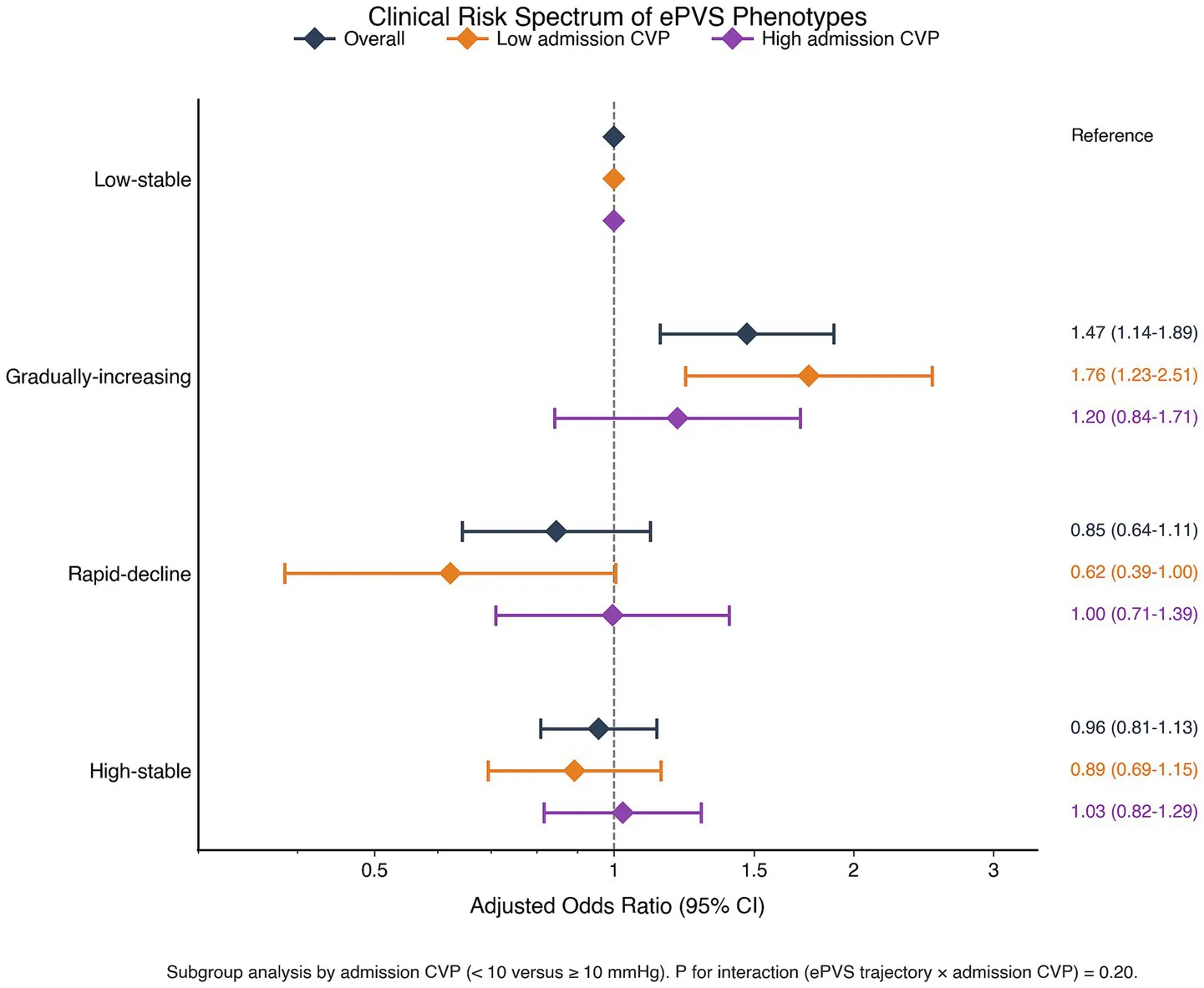
Exploratory central venous pressure (CVP)-stratified risk spectrum of estimated plasma volume status (ePVS) phenotypes (adjusted OR, Model 3), shown overall and within low (< 10 mmHg) and high (≥ 10 mmHg) admission-CVP strata. The odds ratio for the Gradually-increasing phenotype was higher in the low-CVP stratum (OR = 1.76, 95% CI 1.23 to 2.51) than in the high-CVP stratum (OR = 1.20, 95% CI 0.84 to 1.71), but the trajectory by CVP interaction was not significant (*p* = 0.20). This pattern is therefore exploratory.

In an exploratory CVP-stratified analysis (Figure 4), the Gradually-increasing phenotype was significantly associated with AKI among patients with low admission CVP (< 10 mmHg; OR 1.76, 95% CI 1.23 to 2.51; *p* = 0.002) but not among those with high CVP (≥ 10 mmHg; OR 1.20, 0.84 to 1.71; *p* = 0.31). The trajectory by CVP interaction was not significant (*p* = 0.20), so this pattern is hypothesis-generating. Together, the orthogonality of the two indices, the persistence of the effect after CVP adjustment, and the small, mediated fraction indicate that the dynamic ePVS signal conveys AKI risk that is largely independent of CVP and present even when early venous pressure appears reassuring.

### 3.4 Forward-time landmark analysis amplifies the gradually-increasing risk signal

**Figure 5:**
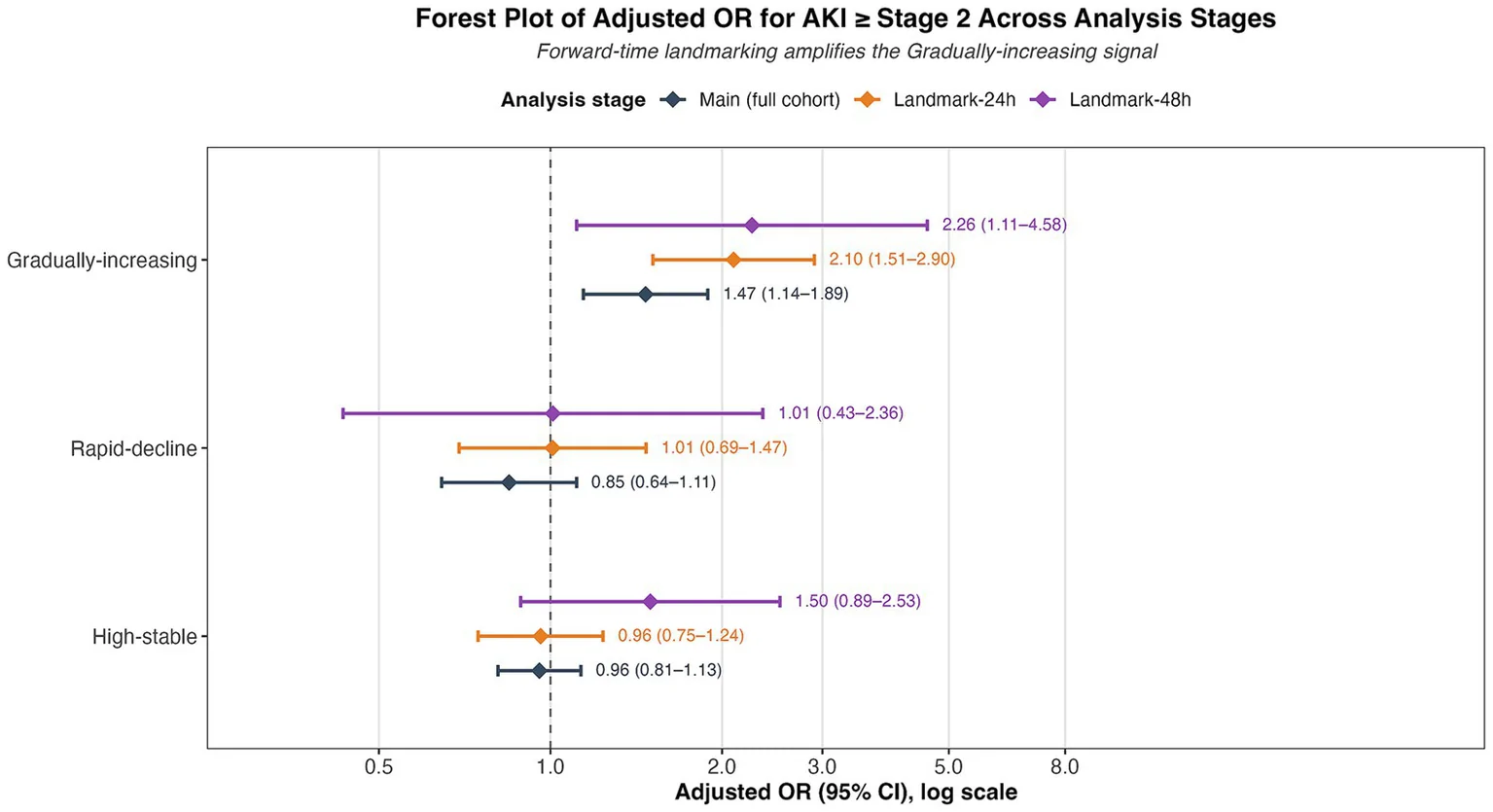
Forest plot of phenotype-specific adjusted odds ratio (OR) for acute kidney injury (AKI) ≥ stage 2 across the full-cohort main analysis, the 24-h landmark, and the 48-h landmark. Strengthening of the gradually-increasing effect under landmarking argues against reverse causation.

Of the 4,818 patients, 20 whose recorded AKI onset preceded ICU admission were excluded from the landmark analyses, leaving 4,798; of these, 1,351 had developed AKI before 24 h (28.2%) and were excluded from the 24-h landmark cohort, leaving 3,447 at risk with 562 incident events between 24 and 72 h (Figure 5). After this restriction, the adjusted OR for the Gradually-increasing phenotype rose from 1.47 in the main analysis to 2.10 (95% CI 1.51 to 2.90; *p* < 0.001), arguing against reverse causation. Strengthening under landmarking argues against reverse causation but may also partly reflect depletion of susceptible patients, so the landmark estimates apply specifically to patients who are event-free at the landmark. Results for the Rapid-decline (OR 1.01, 0.69 to 1.47) and High-stable (0.96, 0.75 to 1.24) phenotypes remained non-significant. At the 48-h landmark (*n* = 2,983 at risk, 98 events), the Gradually-increasing signal persisted in the same direction and magnitude (OR 2.26, 1.11 to 4.58; raw *p* = 0.024), although with only 98 events it was attenuated to borderline after multiple-testing correction (Benjamini-Hochberg *q* = 0.073, Bonferroni-corrected *p* = 0.219; Supplementary Table S4) and should be regarded as supportive rather than confirmatory. Left-truncated Cox models gave hazard ratios of the same direction and magnitude (Supplementary Table S6).

### 3.5 Dynamic trajectory class adds modest incremental value over static admission ePVS

**Figure 6:**
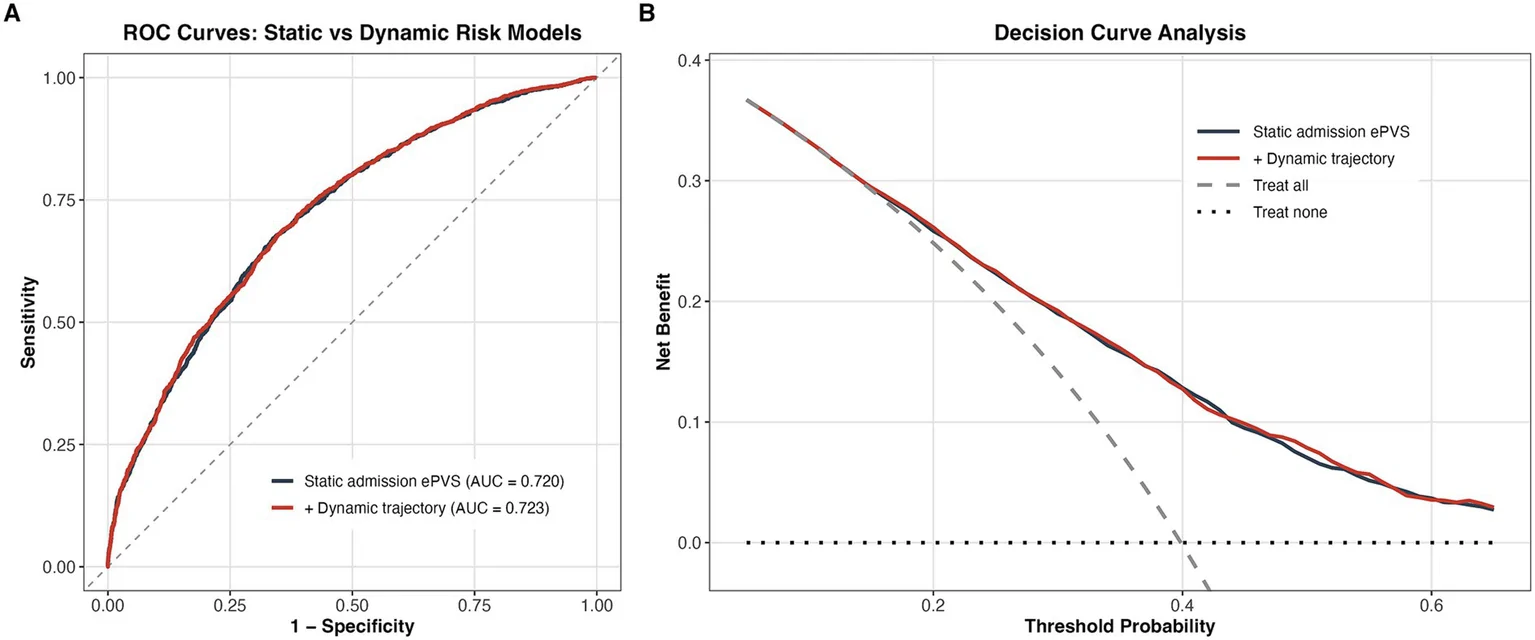
Incremental value of dynamic trajectory information over static admission estimated plasma volume status (ePVS). **(A)** Receiver operating characteristic (ROC) curves for the static and dynamic models. Bootstrap confidence intervals (CIs) were calculated for the change in area under the curve (ΔAUC), net reclassification improvement (NRI), and integrated discrimination improvement (IDI). **(B)** Decision-curve analysis; the static and dynamic models track closely.

A model with admission ePVS plus the Model 3 covariates achieved an AUC of 0.720. Adding trajectory class produced a small but significant improvement: ΔAUC = 0.003 (95% CI 0.001 to 0.006), continuous NRI = 9.9% (2.8 to 16.8%), and IDI = 0.4% (0.1 to 0.8%) (Figure 6A). Decision-curve analysis (Figure 6B) showed the two models tracking closely across all clinically relevant thresholds, with no visible separation in net benefit. Model calibration was adequate (Supplementary Figure S3). On its own, single-time-point ePVS discriminated AKI only weakly (area under the curve, 0.50–0.54 across the four windows; Supplementary Table S7); however, this does not preclude the potential prognostic value of its temporal trajectory. The continuous 0 to 24 h change in ePVS, which can be computed for any patient from two routine blood counts, discriminated no better (area under the curve 0.50; Supplementary Table S7). The Gradually-increasing phenotype could itself be recognized from simple ePVS-derived measures (area under the curve about 0.89 for class membership; Supplementary Table S8), indicating an interpretable pattern rather than an opaque cluster, although this recognition did not translate into AKI discrimination.

## 4 Discussion

In a large CABG cohort, we identified four dynamic ePVS phenotypes, of which the Gradually-increasing trajectory was consistently associated with higher subsequent AKI risk. The prognostic information from ePVS was largely independent of and complementary to that from CVP: admission ePVS and CVP showed minimal correlation (Spearman *r* = 0.04), the phenotype association with AKI was essentially unchanged after CVP adjustment, and peak CVP mediated less than 10% of the total effect. Thus, ePVS conveys plasma-volume information beyond venous pressure. A Gradually-increasing trajectory remained associated with higher AKI risk, even among patients with low CVP, suggesting that serial ePVS may inform postoperative fluid management.

The phenotypes are physiologically interpretable. The Gradually-increasing phenotype began with a near-normal admission ePVS (about 6.6 mL/g) but expanded to a peak near 9.1 mL/g around 36 h; its AKI association strengthened rather than attenuated under forward-time landmarking (OR 1.47, then 2.10, then 2.26), arguing for a true antecedent role. The Rapid-decline phenotype arrived with the highest baseline ePVS but contracted rapidly toward normal and received the largest median transfusion in this group. It was not independently associated with AKI, and this should not be read as transfusion neutralizing harm; more likely, it reflects its distinct, higher-risk baseline profile. The High-stable phenotype, persistently elevated but non-progressive, may represent a chronically expanded but compensated state. These shapes may be understood within the framework of stressed and unstressed intravascular volume (17): a progressively rising ePVS could reflect overflow of the unstressed reservoir as crystalloid accumulates beyond venous capacitance, whereas a rapidly falling ePVS is consistent with active restoration through transfusion or diuresis (18). This interpretation is inferential, and the four phenotypes likely represent regions along a continuum rather than discrete mechanisms. ePVS reflects plasma volume and hemodilution rather than venous tone or compliance, which are the primary determinants of stressed volume, so this framework is interpretive rather than directly measured here.

That CVP explained so little of the ePVS effect is itself the strongest evidence for complementarity. ePVS is neither a measure of total body fluid nor strictly of intravascular volume, but a derived signal of plasma-volume status, integrating intravascular filling (19), capillary integrity (20), and the partition of fluid between vascular and interstitial compartments (21). The large unexplained component may reflect post-bypass injury that pressure metrics do not capture, systemic inflammatory activation with capillary leak (22), endothelial glycocalyx shedding that raises ePVS without raising CVP, microcirculatory renal venous congestion (23), and intra- and post-operative renal medullary hypoxia (24, 25). The exploratory concentration of the ePVS signal in patients with low admission CVP, although the interaction was not significant, is consistent with this picture: where an early venous pressure is reassuring, a rising plasma-volume signal may be precisely the information a clinician would otherwise miss. This hypothesis warrants prospective testing.

Our framing connects to the wider move toward multimodal congestion assessment. Point-of-care venous excess ultrasound (VExUS) characterizes the downstream expression of systemic venous congestion and is linked to AKI in cardiac-surgery and general-ICU cohorts (26) but requires equipment and operator skill. ePVS offers a scalable, near-zero-cost, non-invasive volume axis from routine laboratory data, deployable across diverse services including resource-limited settings. We do not propose ePVS as a replacement for pressure- or ultrasound-based assessment but as a complementary signal adding the plasma-volume dimension absent from CVP (7). Other routinely available markers describe different axes of the same syndrome. In a single-center series of 186 CABG patients, the C-reactive protein to albumin ratio predicted postoperative AKI with an area under the curve of 0.78 (27), an inflammatory signal rather than a volume one. An assessment spanning inflammation, venous pressure, and plasma volume may characterize risk better than any single axis. As latent-class phenotyping has revealed treatment-responsive subgroups in sepsis and acute respiratory distress syndrome (28), a rising ePVS in an apparently low-risk patient may signal an evolving congestion process that could still be aborted by reassessing maintenance fluids, restrictive fluid strategies, early diuresis or, in selected patients, ultrafiltration, and the structured KDIGO care bundle shown to reduce moderate-to-severe CSA-AKI (29). Contemporary postoperative CABG care already emphasizes goal-directed therapy and enhanced recovery protocols (30), into which a volume trajectory derived from routine blood counts could be incorporated without additional measurement.

This study has strengths: a large cohort, a fully reproducible pipeline, latent-class modeling of raw longitudinal data rather than discretized snapshots, and a layered design integrating orthogonality, mechanism, reverse-causation, and incremental-value analyses. Limitations include the single-center nature of MIMIC-IV; the mathematical dependence of ePVS on hemoglobin, so that ePVS partly reflects hemodilution and transfusion despite adjustment; the modest incremental discrimination over a static measurement; the non-significant trajectory by CVP interaction, which means the CVP-stratified pattern is hypothesis-generating; the use in the stratified analysis of a single early (admission) CVP rather than a dynamic CVP comparator, a fairer test that should remain for future work; the absence from MIMIC-IV of intraoperative variables such as cardiopulmonary bypass and aortic cross-clamp duration, which could confound the association; the exploratory nature of the subgroup and mediation analyses, interpreted after multiple-testing correction; the unavailability in MIMIC-IV of advanced congestion and tubular-stress measures and the loss of power under landmark restriction, particularly at 48 h. Finally, the trajectory phenotypes are derived by latent-class modeling and are a descriptive lens on an underlying ePVS continuum rather than a bedside-ready tool: assigning an individual patient to the Gradually-increasing class requires the full longitudinal model, which class comprises only 6.9% of the cohort, and the pattern cannot yet be reduced to a simple threshold or rate-of-rise criterion.

We therefore present ePVS as a continuous trend to be monitored rather than a dichotomous trigger, and the derivation and prospective validation of an operational bedside criterion remain necessary. The clinically meaningful quantity here is the trend, not a single value, and it is especially informative where a reassuring venous pressure would otherwise suggest that volume status is not a concern. Future work includes external validation, prospective testing of ePVS-guided fluid strategies in the Gradually-increasing phenotype, and integration with biomarkers of capillary leak and renal tubular stress.

## 5 Conclusion

Four distinct ePVS trajectories within 48 h after CABG carry differential AKI risk. The Gradually-increasing phenotype was independently associated with AKI, and its association strengthened under forward-time validation. This risk was largely independent of and complementary to central venous pressure, adding a plasma-volume axis that pressure-based monitoring does not capture. Prospective, phenotype-targeted studies are needed to determine whether acting on this trajectory improves outcomes.

## Data Availability

The original contributions presented in the study are included in the article/Supplementary material, further inquiries can be directed to the corresponding author.
