# Supplementary material for: Association between dynamic estimated plasma volume trajectories and acute kidney injury after coronary artery bypass grafting
Source: Front Med (Lausanne). 2026 Sep 9;13:1912347. doi: 10.3389/fmed.2026.1912347 (PMC13597669; doi:10.3389/fmed.2026.1912347)
Supplement: Supplementary file 1 [file Table_1.docx]

**Supplementary Table S1.** Pre-imputation missingness for the analytic variables.

| **Variable** | **Missing n** | **Missing %** |
| --- | --- | --- |
| ePVS 0-6 h | 6 | 0.1 |
| ePVS 12 h | 197 | 4.1 |
| ePVS 24 h | 92 | 1.9 |
| ePVS 48 h | 462 | 9.6 |
| Admission CVP | 63 | 1.3 |
| 12-h CVP | 284 | 5.9 |
| BMI | 113 | 2.3 |
| All other Model-3 covariates | 0 | 0.0 |

*Complete-case n = 3,962 (82.2%). Missing ePVS/CVP values (and IQR-flagged extremes, k=2.0) were imputed by predictive mean matching (mice).*

**Supplementary Table S2.** Latent-class trajectory model selection (1-5 classes) and stability of the high-risk phenotype.

| **Number of classes** | **BIC** | **Smallest class (%)** | **Highest-AKI class: baseline→peak ePVS (AKI %)** |
| --- | --- | --- | --- |
| 1 | 125,643 | 100.0 | — |
| 2 | 122,856 | 17.8 | — |
| 3 | 122,482 | 4.6 | low-rising 6.15→8.70 (48.0%) |
| 4 (selected) | 122,108 | 6.9 | low-rising 6.56→9.09 (47.0%) |
| 5 | 122,065 | 4.5 | low-rising 6.20→8.77 (49.6%) |

*The BIC decreased monotonically with additional classes; the four-class solution was selected on the basis of entropy, an adequate minimum-class proportion, and clinical interpretability (four-class mean posterior probability 0.83). A low-baseline, progressively-rising phenotype carrying the highest AKI rate was present in every solution from three to five classes, confirming that the high-risk phenotype is not an artefact of the chosen class number. Latent-class linear mixed model (lcmm::hlme): class-specific quadratic fixed time effect, random intercept, gridsearch with random starts.*

**Supplementary Table S3.** Sensitivity of the Gradually-increasing vs Low-stable association to the depth of covariate adjustment (over-adjustment / collider check).

| **Model** | **OR (95% CI)** | **p** |
| --- | --- | --- |
| Crude (unadjusted) | 1.35 (1.08-1.70) | 0.010 |
| Pre-operative confounders only | 1.56 (1.22-2.00) | 0.001 |
| Model 1 (+ SOFA) | 1.56 (1.21-2.00) | 0.001 |
| Model 3 (full) | 1.44 (1.11-1.85) | 0.005 |
| Complete-case Model 3 (no imputation, n=3,962) | 1.43 (1.09-1.88) | 0.009 |

*The association is significant unadjusted and stable across adjustment depths and in a complete-case analysis, indicating it is not an artefact of adjustment or of imputation.*

**Supplementary Table S4.** Multiple-testing correction for the primary family of phenotype comparisons (Benjamini-Hochberg FDR and Bonferroni).

| **Comparison** | **Raw p** | **BH q** | **Bonferroni p** |
| --- | --- | --- | --- |
| Main analysis, Gradually-increasing | 0.003 | 0.012 | 0.024 |
| Main analysis, Rapid-decline | 0.228 | 0.410 | 1.000 |
| Main analysis, High-stable | 0.600 | 0.900 | 1.000 |
| 24-h landmark, Gradually-increasing | <0.001 | <0.001 | <0.001 |
| 24-h landmark, Rapid-decline | 0.965 | 0.982 | 1.000 |
| 24-h landmark, High-stable | 0.756 | 0.972 | 1.000 |
| 48-h landmark, Gradually-increasing | 0.024 | 0.073 | 0.219 |
| 48-h landmark, Rapid-decline | 0.982 | 0.982 | 1.000 |
| 48-h landmark, High-stable | 0.131 | 0.295 | 1.000 |

*The Gradually-increasing association remains significant after correction in the main and 24-h landmark analyses; the 48-h landmark is attenuated to borderline owing to event sparsity.*

**Supplementary Table S5.** Parallel multiple-mediator decomposition of the admission-ePVS effect on AKI, with proportion mediated and 95% CIs.

| **Mediator** | **Indirect effect a x b (95% CI)** | **Proportion mediated (95% CI)** | **Significance** |
| --- | --- | --- | --- |
| Delta Hgb (0-24 h) | 0.014 (-0.022 to 0.052) | 43.0% (-67% to 158%) | Not significant (CI crosses zero) |
| Peak CVP (0-24 h) | 0.003 (0.001 to 0.006) | 9.9% (3.0% to 18.2%) | Significant |
| Net fluid balance (0-24 h) | 0.004 (0.0003 to 0.0072) | 10.7% (1.0% to 21.8%) | Significant |

*Total effect on the logit scale = 0.033. Proportion-mediated CIs are derived from the bootstrap indirect-effect CIs relative to the total effect. Because ePVS is a deterministic function of haemoglobin, the Delta Hgb pathway is not statistically identifiable and its estimate should not be interpreted.*

**Supplementary Table S6.** Left-truncated Cox proportional-hazards models for the landmark analyses: phenotype-specific hazard ratios for incident AKI ≥ stage 2.

| **ePVS trajectory phenotype** | **24-h landmark HR (95% CI), p** | **48-h landmark HR (95% CI), p** |
| --- | --- | --- |
| Low-stable | Reference | Reference |
| Gradually-increasing | 1.87 (1.43 to 2.46), <0.001 | 2.24 (1.14 to 4.42), 0.020 |
| Rapid-decline | 1.04 (0.75 to 1.44), 0.810 | 1.05 (0.47 to 2.38), 0.901 |
| High-stable | 0.97 (0.78 to 1.21), 0.792 | 1.50 (0.90 to 2.48), 0.118 |

Left-truncated (delayed-entry) Cox models entered at each landmark; patients with AKI ≥ stage 2 before the landmark were excluded, and the outcome was incident AKI ≥ stage 2 between the landmark and 72 h. Hazard ratios are adjusted for the Model 3 covariates (age, sex, body mass index, SOFA score, congestive heart failure, diabetes without complications, chronic pulmonary disease, first-24-h mean arterial pressure, peak lactate, admission CVP, 0 to 24 h red blood cell transfusion volume, and noradrenaline use). Low-stable is the reference. 24-h landmark: 3,447 at risk, 562 events; 48-h landmark: 2,983 at risk, 98 events. Hazard ratios are of the same direction and magnitude as the corresponding logistic odds ratios (Figure 5).

**Supplementary Table S7.** Discrimination of single-time-point ePVS and the 0 to 24 h ePVS change, the 0 to 48 h change, and the rate of change for AKI >= stage 2 (ROC / Youden). Acute kidney injury rates across strata of admission ePVS are also shown below.

| **Marker** | **AUC** | **Youden cut-off (mL/g)** | **Sensitivity** | **Specificity** |
| --- | --- | --- | --- | --- |
| ePVS 0-6 h | 0.518 | 5.01 | 0.86 | 0.17 |
| ePVS 12 h | 0.499 | 6.77 | 0.46 | 0.56 |
| ePVS 24 h | 0.535 | 7.11 | 0.57 | 0.50 |
| ePVS 48 h | 0.532 | 6.94 | 0.69 | 0.37 |
| Peak ePVS 0-24 h | 0.519 | 8.73 | 0.35 | 0.69 |
| ePVS change 0-24 h | 0.504 | +0.44 | 0.47 | 0.55 |
| ePVS change 0-48 h | 0.505 | +0.52 | 0.52 | 0.50 |
| ePVS rate of change 0-24 h | 0.504 | +0.024 | 0.47 | 0.55 |
| ePVS rate of change 0-48 h | 0.505 | +0.012 | 0.52 | 0.50 |

*Single-point ePVS discriminates AKI only weakly (AUC 0.50-0.54) with no clinically useful Youden threshold, supporting its use as a complementary risk signal rather than a stand-alone diagnostic cut-off.* Rate-of-change cut-offs are expressed in mL/g per hour; because the rate is the fixed-window change divided by a constant, its discrimination equals that of the corresponding change.

**Acute kidney injury (≥ stage 2) rate by stratum of admission ePVS.**

| **Stratum** | **n** | **AKI ≥ stage 2 (%)** |
| --- | --- | --- |
| Admission ePVS quintile 1 (lowest) | 964 | 36.5 |
| Quintile 2 | 965 | 42.1 |
| Quintile 3 | 969 | 40.0 |
| Quintile 4 | 957 | 39.3 |
| Quintile 5 (highest) | 963 | 42.7 |
| Admission ePVS ≤ 7 mL/g | 2,603 | 39.8 |
| Admission ePVS > 7 mL/g | 2,215 | 40.5 |

*AKI rates are crude. No consistent gradient was seen across quintiles of admission ePVS (trend p = 0.06 unadjusted, p = 0.28 after covariate adjustment). Admission ePVS above versus at or below 7 mL/g gave an adjusted odds ratio of 0.98 (95% CI 0.85 to 1.12, p = 0.74). No stratum of absolute ePVS identified a group at materially higher AKI risk.*

**Supplementary Table S8.** Identification of the Gradually-increasing trajectory by simple ePVS-derived measures.

| **Measure** | **AUC for Gradually-increasing class membership** |
| --- | --- |
| Peak minus admission ePVS | 0.90 |
| 0 to 24 h change | 0.89 |
| 24 h ePVS | 0.88 |
| 0 to 48 h change | 0.83 |
| Peak ePVS | 0.80 |

*A transparent rule (peak rise ≥ 2 mL/g with admission ePVS ≤ 7.5 mL/g) captured about 80% of the Gradually-increasing class (n = 332; 6.9% of the cohort). Because the class is uncommon and its excess risk is modest (AKI 47% versus 40% in the remainder), the rule flagged 1,242 patients, of whom about one in five belonged to the class, and this group had an AKI ≥ stage 2 rate of 40.3%, similar to the cohort as a whole (40.1%). The trajectory is identifiable but does not yield a bedside risk threshold. AUC, area under the receiver-operating-characteristic curve for identifying class membership.*
